# Supplementary material for: Weaker neural suppression in autism
Source: Nat Commun. 2020 May 29;11:2675. doi: 10.1038/s41467-020-16495-z (PMC7260360; doi:10.1038/s41467-020-16495-z)
Supplement: Supplementary file 1 — Supplementary Information [file 41467_2020_16495_MOESM1_ESM.pdf]

Supplementary Information

Weaker neural suppression in autism

Schallmo, et al. (2020)

Nature Communications

Supplementary Notes

After observing weaker neural suppression in the foveal hMT+ fMRI response in participants with ASD, we asked whether this difference in suppression might be attributable to weaker inhibition, consistent with theories of E/I imbalance in this disorder[1, 2, 3]. To attempt to address this question, we used MR spectroscopy to measure the concentration of GABA+ (GABA, an inhibitory neurotransmitter, plus co-edited macromolecules) in a region centered around hMT+ in the lateral occipital lobe (Supplementary Figure 1B; Supplementary Figure 2A & B). We predicted that if weaker spatial suppression was driven by reduced inhibition in hMT+, this might be reflected in lower GABA+ levels as measured by MRS. However, we found no significant difference in GABA+ levels within the MT region between participants with ASD versus NTs (main effect of group, $F_{1, 60} = 1.21$, $p = 0.3$; Supplementary Figure 2C). We also found no significant correlations between GABA+ levels in hMT+ and measures of suppression (size indices or fMRI; averaging across low & high contrast conditions to minimize multiple comparisons; correlations for both groups combined: $|r_{47\text{-}60}| < 0.10$, uncorrected p-values > 0.5; for ASD participants alone: $|r_{22\text{-}25}| < 0.28$, uncorrected p-values > 0.19; for NTs alone: $|r_{23\text{-}33}| < 0.21$, uncorrected p-values > 0.3; Supplementary Figure 2E & F). This agrees with our recent findings from a subset of these NTs[5]. Although these results do not lend support to the hypothesis that weaker spatial suppression in ASD may be attributed to differences in the level of GABA+ in hMT+, the lack of a significant relationship precludes us from drawing any strong conclusions about the role of GABA+ in spatial suppression among people with ASD.

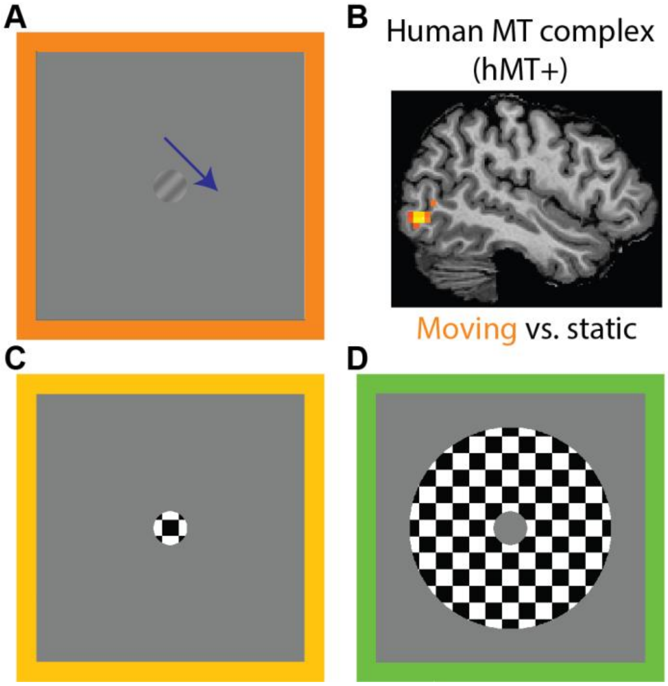

Supplementary Figure 1. Functional localizers. **A)** Functional localizer scan #1 included blocks of moving vs. static gratings. **B)** Human MT complex (hMT+) was identified in the lateral occipital lobe using a correlational analysis (motion > static; yellow voxels). Functional localizer scan #2 included blocks of flickering checkerboards in the central 2° **(C)**, vs. surrounding 12° **(D)**. Foveal hMT+ ROIs were defined by (motion > static) & (center > surround).

Our MRS procedure also allowed us to measure a signal associated with glutamate, an excitatory neurotransmitter. Because this signal also includes some contribution from glutamine and glutathione[6], we refer to this measure as Glx. Some previous studies have suggested that surround suppression is driven primarily by a withdrawal of excitation, rather than inhibition of neural activity[7, 8]. There is also limited evidence suggesting increased glutamatergic excitation in ASD[9, 10]. Therefore, we examined whether weaker suppression in ASD might be related to differences in excitatory neurotransmitter levels, which could be reflected in the Glx signal from the hMT+ region. However, we observed no significant difference between groups in Glx within hMT+ (main effect of group; $F_{1, 60} = 1.54$, $p = 0.2$; Supplementary Figure 2D). Further, Glx levels in hMT+ did not correlate significantly with behavioral or fMRI measures of suppression (correlations for both groups combined: $|r_{47\text{-}60}| < 0.06$, uncorrected p-values > 0.6; ASD participants alone: $|r_{22\text{-}25}| < 0.28$, uncorrected p-values > 0.18; NTs alone: $|r_{23\text{-}33}| < 0.21$, uncorrected p-values > 0.3; Supplementary Figure 2G & H). Thus, we found no evidence for an association between Glx levels in hMT+ and spatial suppression, and we cannot form a strong conclusion regarding the role of glutamatergic excitation in ASD.

As MRS data were acquired in both left and right hMT+ in separate scans, we compared data between hemispheres to examine potential lateralized differences in metabolite concentrations, or interactions between participant group and hemisphere. We found no difference in GABA+ levels between left and right hMT+ across groups (GABA+: left mean = 3.25 i.u., $SD = 0.23$; right mean = 3.24 i.u., $SD = 0.29$; main effect of hemisphere, $F_{1, 60} = 0.02$, $p = 0.9$). In contrast, Glx levels were higher in right as compared to left hMT+ (Glx: left mean = 5.51 i.u., $SD = 0.90$, right mean = 6.70 i.u., $SD = 0.69$; main effect of hemisphere, $F_{1, 60} = 103$, $p = 1 \times 10^{-14}$). However, there were no significant interactions between group & hemisphere for either metabolite ($F_{1, 60} < 1.59$, p-values > 0.2).

Because our GABA+ values were corrected for tissue fraction, while our Glx values were not (see Methods), we

asked whether differences in gray matter, white matter, and CSF fractions within the voxel might have contributed to the observed difference in Glx levels between left and right hMT+. We found that the proportion of CSF was indeed higher within left vs. right hMT+ voxels (left mean = 3.79%, *SD* = 1.99%; right mean = 3.30%, *SD* = 1.54%; $F_{1, 60}$ = 8.77, p = 0.004). There were no significant differences in gray or white matter content between hemispheres (GM: left mean = 44.5%, *SD* = 3.67%; right mean = 44.3%, *SD* = 3.24%; $F_{1, 60}$ = 0.14, p = 0.7; WM: left mean = 51.7%, *SD* = 4.22%; right mean = 52.4%, *SD* = 3.51%; $F_{1, 60}$ = 2.64, p = 0.11). Differences in CSF were reflected in the water reference; the measured water signal was also higher in left vs. right hMT+ (left mean = 30.5 i.u., *SD* = 5.33, right mean = 28.9 i.u., *SD* = 5.46; $F_{1, 60}$ = 27.4, p = 2 x 10^{-6}). However, there were no significant differences between participants with ASD and NTs in gray matter, white matter, CSF, or water levels ($F_{1, 60}$ < 3.54, uncorrected *p*-values > 0.065), and no significant interactions between group and hemisphere ($F_{1, 60}$ < 2.25, uncorrected *p*-values > 0.139). Thus, although differences in Glx values from left vs. right hMT+ depended on CSF fraction and the associated water reference signal, we did not find evidence to suggest that these effects were associated with any differences in MRS data between groups.

To further examine whether our MRS results in hMT+ were affected by scaling our metabolite values (GABA+ and Glx) relative to water, we repeated our analyses using values instead scaled relative to creatine. We found comparable results for the creatine scaled data; there were no group differences in GABA+ ($F_{1, 60}$ = 1.42, p = 0.2) or Glx ($F_{1, 60}$ = 0.84, p = 0.4), and no correlations with suppression metrics (size indices or fMRI suppression, averaged across contrast conditions to minimize multiple comparisons, $|r_{47\text{-}60}|$ < 0.16, uncorrected *p*-values > 0.2). Thus, we found no evidence to suggest that GABA+ or Glx levels in hMT+, as measured by MRS, differed between participants with ASD and NT controls.

Additional MRS data were acquired in a mid-occipital voxel within early visual cortex (EVC; Supplementary Figure 3A & B). We first examined whether metabolite values (GABA+ or Glx) in EVC differed between groups. No significant group differences were observed (main effect of group, GABA+: $F_{1, 60}$ = 0.52, p = 0.5; Glx: $F_{1, 60}$ = 0.69, p = 0.4; Supplementary Figure 3C & D). We also examined correlations between GABA or Glx in EVC and size indices from our psychophysical task, none of which were significant (correlations examined both within and across participant groups, size indices averaged across contrast conditions to

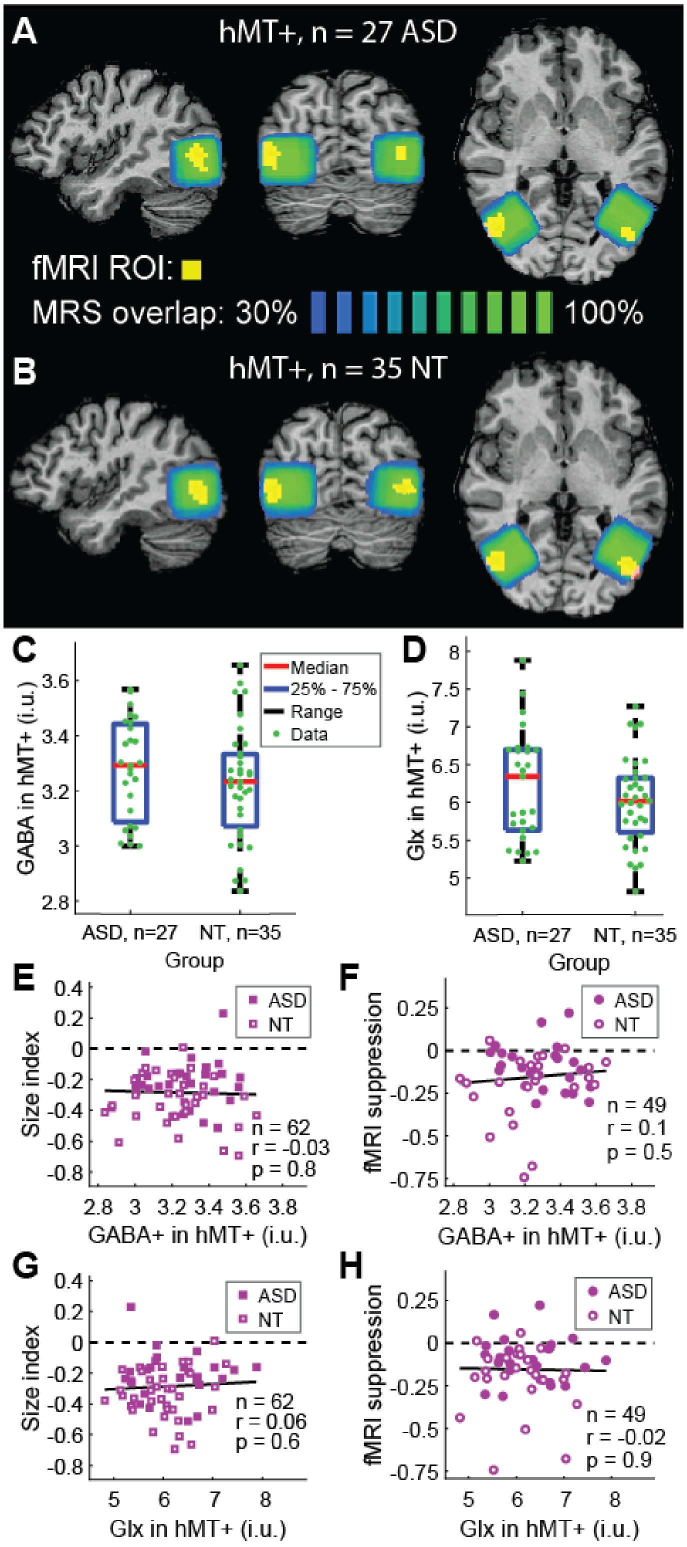

Supplementary Figure 2. MR spectroscopy results in hMT+. **A)** Voxel positions for left and right hMT+ in Talairach space for n = 27 participants with ASD. Blue-green color indicates overlap across individuals. Yellow indicates average hMT+ ROI across participants, from fMRI (correlation of predicted vs. observed response, thresholded at Pearson's r_{123} = 0.25). **B)** Same, but for n = 35 NTs. **C)** GABA+ concentrations in hMT+ (averaged left and right voxels in each participant) in institutional units (i.u.). **D)** Same, but for Glx. Correlation (Pearson's) between GABA+ in hMT+ and **E)** size indices or **F)** fMRI suppression (low & high contrast averaged). **G & H)** Same, but for Glx.

minimize multiple comparisons, $|r_{25\text{-}60}| < 0.23$, uncorrected p-values > 0.19). Thus, as in hMT+, we did not find clear evidence of an influence of GABA+ or Glx levels in EVC during spatial suppression in ASD, and cannot form any strong conclusions regarding the possible role(s) of these metabolites in this phenomenon and/or disorder.

To reconcile our current modeling work with previous studies[11, 12], we asked whether spatially narrower top-down modulation within the normalization model might be sufficient to account for previous findings of lower[4] or higher[12] motion duration thresholds in ASD. Indeed, we found that using an overall narrower set of top-down modulation parameters (2 vs. 6 arbitrary units) yielded model predictions (Supplementary Figure 4A-C) that showed a good qualitative match to the results of Foss-Feig and colleagues[4]. Similar to our model predictions, they observed smaller duration thresholds across stimulus sizes for high contrast gratings, and for large, low contrast stimuli in participants with ASD. Their results had previously been modeled by Rosenberg and colleagues[11] using weaker divisive normalization. However, weaker normalization was not sufficient to account for our observation of weaker spatial suppression in ASD, as reducing normalization did not dramatically affect size indices (red arrows, Figure 5A-C). Thus, our narrower top-down modulation model is capable of describing our own behavioral results (Figure 2A-C) as well as the findings of Foss-Feig and colleagues[4] (Supplementary Figure 4A-C), whereas a weaker normalization model[11] is suited only for the latter case.

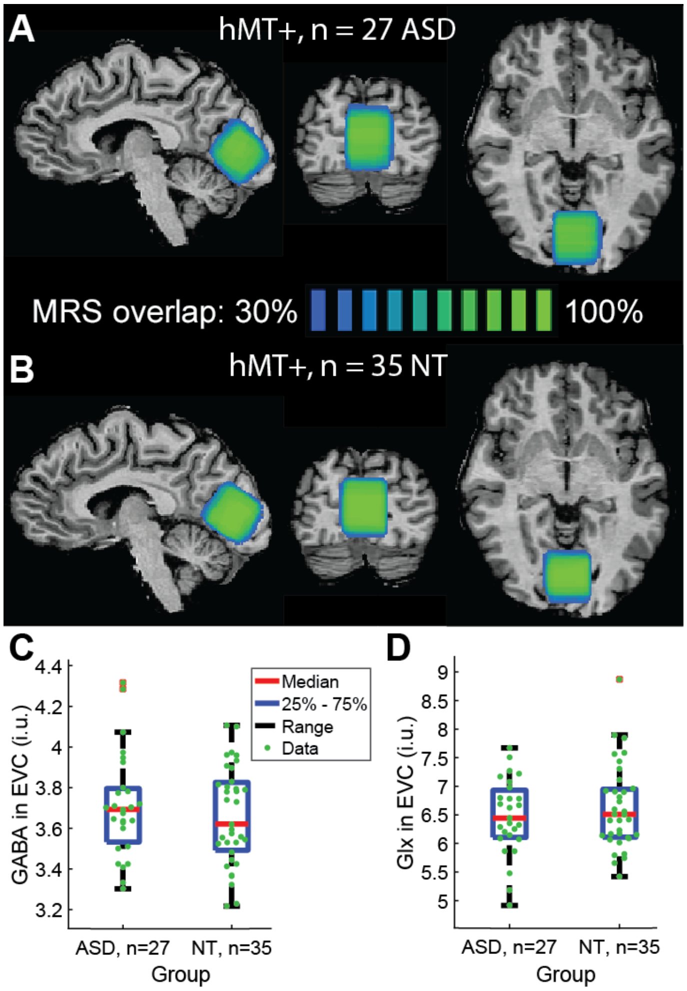

Supplementary Figure 3. MRS results in EVC. **A)** Average voxel positions for EVC in Talairach space for n = 27 participants with ASD. Blue-green color indicates percent overlap across individuals. **B)** Same, but for n = 35 NT participants. **C)** GABA+ concentrations in EVC. Values are in institutional units (i.u.). **D)** Same, but for Glx.

Additionally, we found a different pattern of results using an even narrower top-down modulation field (1 vs. 6 arbitrary units). Model duration thresholds differed less at high contrast, and were actually higher at low contrast for very-narrow vs. broader top-down modulation in our model (Supplementary Figure 4D-F). This occurs because the top-down modulation field is actually narrower than the response region that is read out (i.e., averaged when computing the predicted threshold; r_w; Supplementary Equation 5). Schauder and colleagues[12] found either equivalent or higher duration thresholds for participants with ASD (depending on stimulus condition; see also [13]), and modeled this in terms of an increase in the width of excitatory spatial filters. Although we found that wider excitatory spatial filters yielded weaker spatial suppression, the pattern of duration thresholds predicted by this model did not match our observations in participants with ASD (model duration thresholds were higher for small stimuli, rather than lower for large stimuli; red arrows, Figure 5D-F).

Spatially narrower top-down gain modulation may offer a parsimonious explanation for the difference in motion discrimination duration thresholds in ASD vs. NT participants. We found this computational approach was sufficient to model both the pattern of results we observed in the current study (Figure 5D G-I) and those of previous studies[4, 12, 13] (Supplementary Figure 4), while previous models[11, 12] were not sufficient to describe our observation of weaker spatial suppression in ASD. In Supplementary Figure 5, we provide a more thorough comparison of predictions across model variants. This figure illustrates that in the model proposed by Rosenberg and colleagues[11], reducing normalization does not yield weaker spatial suppression (red arrows, Supplementary Figure 5D). Likewise, we show that increasing the width of excitatory spatial filters, as suggested by Schauder and colleagues[12], predicts higher duration thresholds for small stimuli, rather than lower thresholds for larger gratings (red arrows, Supplementary Figure 5E & F).

Previous studies have suggested that age[14, 15, 16], biological sex[17], and IQ[17, 18, 19] may each influence motion discrimination thresholds. None of these demographic factors differed significantly between our groups of participants with ASD and NTs (Table 1). Nevertheless, to further control for these factors, we conducted post-hoc analyses to examine group differences in duration thresholds, size indices, and fMRI suppression, with age, sex, and non-verbal IQ included as covariates. In each case, the results reported in the main text were recapitulated in the post-hoc analyses; we saw a significant interaction between group and size in our analysis of motion duration thresholds, and significant group differences for size indices and fMRI suppression in hMT+ (linear mixed-effect models, thresholds: interaction between group and size, parameter estimate [SE]: 3.77 [0.59], t_{1420} = 6.40, p = 2 x 10^{-10}; size indices: main effect of group, parameter estimate [SE]: 0.152 [0.042], t_{497} = 3.60, p = 4 x 10^{-4}; fMRI suppression: main effect of group, parameter estimate [SE]: 0.176 [0.061], t_{93} = 2.91, p = 0.005). These post-hoc results suggest that our observations of weaker suppression during motion discrimination and in the fMRI response within hMT+ among participants with ASD vs. NTs are not explained by subtle differences between groups in demographic factors such as age, sex, or IQ.

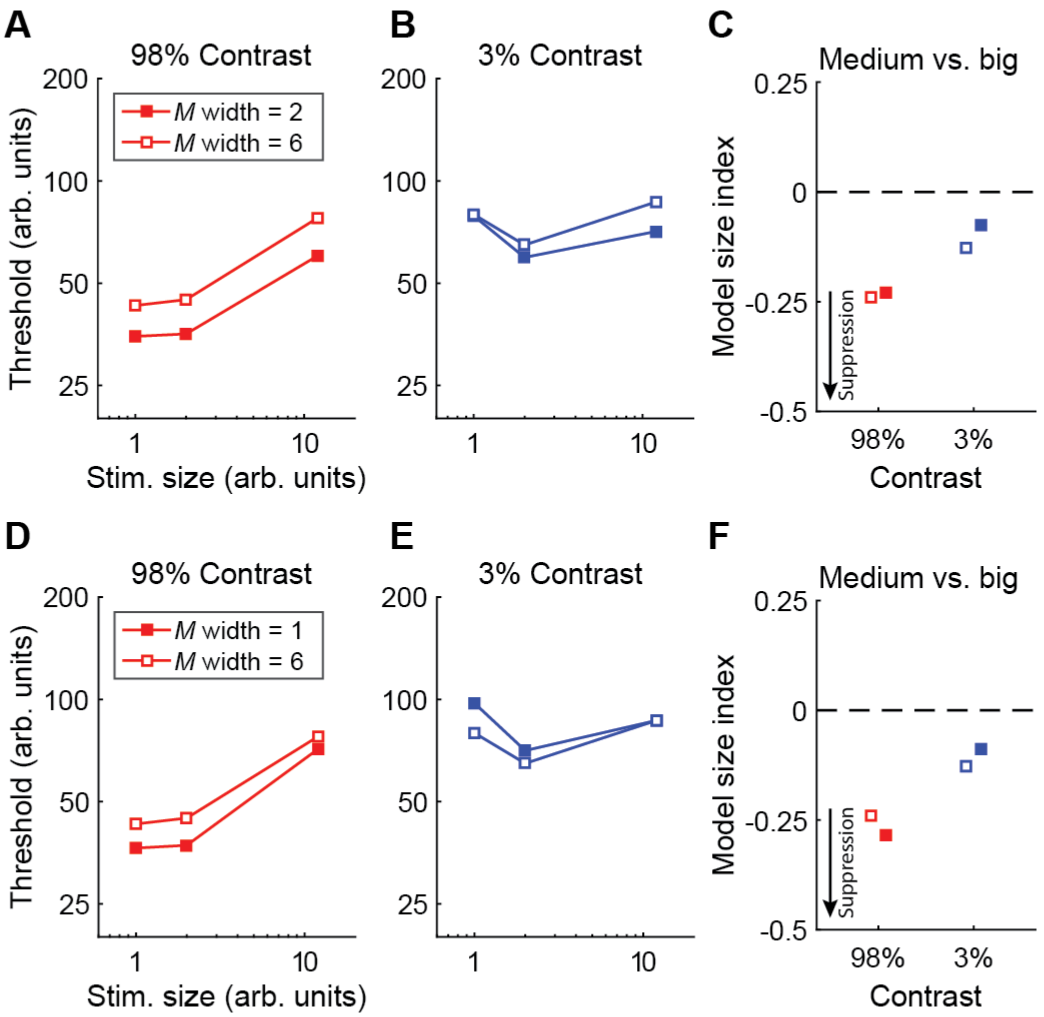

Supplementary Figure 4. Supplementary model results. **A-C)** A narrower set of top-down modulation field parameters (the spatial width of the parameter *M* from Supplementary Equation 1), abbreviated *M* width; 2 vs. 6 arbitrary units) yields model predictions that are reasonably well matched to the observations of Foss-Feig and colleagues[4]; duration thresholds are lower across sizes at high contrast, and for large, low contrast stimuli. **D-F)** Further reducing the width of the narrower top-down modulation (to 1 arbitrary unit) reveals a different, mixed pattern; duration thresholds are lower for high contrast, but higher for low contrast stimuli.

Across both ASD and NT participants there was a significant effect of biological sex on motion duration thresholds, with males showing lower threshold values (mean = 50.3 ms) versus females (mean = 72.4 ms; linear mixed-effect model, main effect of sex, parameter estimate [SE]: 18.1 [5.66], t_{1420} = 3.20, p = 0.001). There was also a significant effect of sex on size indices, such that males showed weaker spatial suppression (mean = 0.25) during psychophysics versus females (mean = 0.34; main effect of sex, parameter estimate [SE]: -0.080 [0.037], t_{497} = -2.17, p = 0.030), but no effect on fMRI suppression in hMT+ (main effect of sex, parameter estimate [SE]: 0.035 [0.047], t_{93} = 0.739, p = 0.5). This agrees with our recent work[17] showing lower duration thresholds among males across multiple experiments (note that the first cohort of participants in our previous study overlaps with the NTs in the current experiments).

Finally, we explored the possible role of demographic factors in our MRS results. There were no significant group differences (NT vs. ASD) in GABA or Glx in either hMT+ or EVC when including age, sex, and IQ as factors in linear mixed

effects models ($|t_{59-119}| < 1.26$, p-values > 0.2), and no significant main effects of sex ($|t_{59-119}| < 1.81$, p-values > 0.075).

We also considered whether our fMRI results showing weaker suppression in hMT+ in participants with ASD might be explained by differences in head motion or a lack of engagement in the color-shape conjunction task (performed at fixation). To this end, we excluded fMRI blocks (10 s) with excessive head motion, and scans (4 min) in which there was poor fixation task performance (see Supplementary Methods for details). Data from 2 ASD participants and 1 NT were completely excluded, as there was not a sufficient amount of data for analysis following the above exclusion. Despite the reduction in sample size, the results of this secondary analysis were qualitatively the same as those reported for the full data set (Figure 2D-F); in response to larger drifting gratings, suppression of the

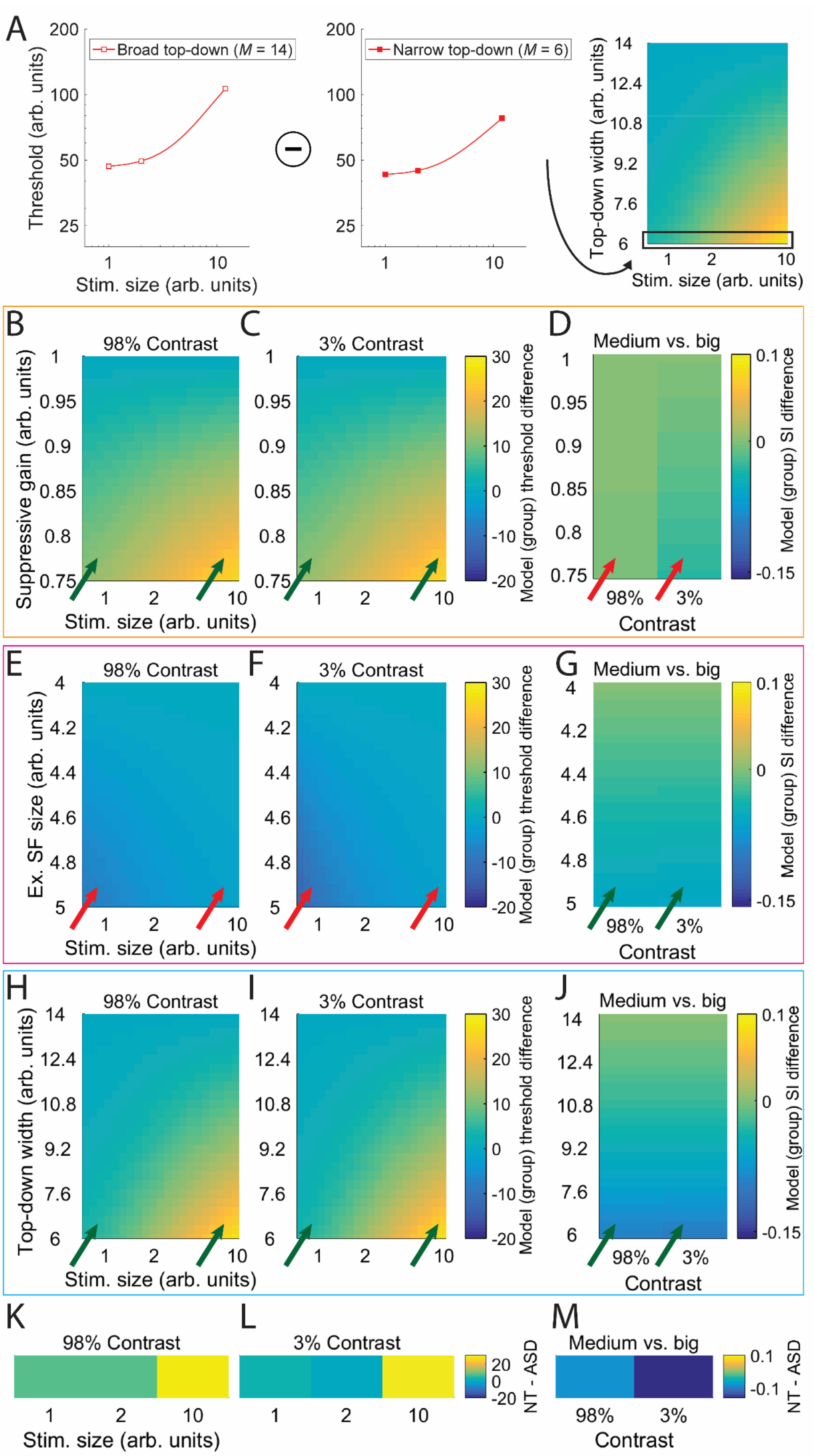

Supplementary Figure 5. Normalization model surfaces. **A)** Diagram showing how model surfaces were generated. The effect of manipulating different model parameters was examined by finding the difference in duration thresholds between the base model (i.e., designed to match NT data; left) and the altered model (i.e., intended to match data from participants with ASD; middle) …

(Supplementary Figure 5, continued). These threshold differences were then plotted within the model surface (black arrow and box). This was done for each parameter value along the y-axis of the model surface. The same subtraction procedure was also performed for size indices. **B-M)** Left & middle panels: difference in predicted thresholds (i.e., group difference) for different parameter values. Right panels: the same for size indices (medium vs. big). Y-axes show the parameter of interest. Differences in thresholds and size indices are calculated relative to the model prediction using the top-most value in each panel (i.e., the base model that shows a good match to data from NTs; Supplementary Table 2). **B-D)** Weaker normalization. **E-G)** Wider excitatory spatial filters. **H-J)** Narrower top-down gain. **K & L)** Average difference in duration thresholds for NT - ASD participants (the actual pattern of behavioral data we observed, as in Figure 2A-B), for comparison. **M)** Same, but for size indices. Colored boxes encompass different model variants, as in Figures 4 & 5. Red arrows in **B-J** show where predictions of these model variants are a poor match to the results we observed in participants with ASD vs. NTs, green arrows indicate a good match (compare with **K-M**). Weak normalization predicts little to no change in size indices (**D**), whereas wider excitatory spatial filters predict higher rather than lower thresholds (a negative difference; **E & F**).

fMRI signal in hMT+ was weaker in participants with ASD (Supplementary Figure 6; ANOVA, main effect of group: $F_{1, 44}$ = 4.10, p = 0.049). This suggests that differences in head motion or fixation task performance between groups may not account for weaker fMRI suppression within hMT+ in ASD.

Further, we examined whether differences in eye movements between groups might account for weaker spatial suppression in our participants with ASD. We conducted a series of analyses to examine relationships between suppression metrics from our behavioral and fMRI experiments, and eye tracking data collected simultaneously. We used 3 eye tracking metrics: mean distance from center (a measure of gaze accuracy in space), the standard deviation of fixation distance from center (a spatial measure of gaze variability), and mean fixation duration (a measure of fixation stability across time). Eye tracking data were drift corrected post-hoc (see Supplementary Methods for eye tracking analysis details). To assess whether fixation behavior may have influenced our observation of weaker spatial suppression in ASD, we examined group differences in eye tracking metrics, and correlations between these metrics and psychophysical or fMRI measures of suppression across both ASD and NT participants.

Eye tracking data were obtained during psychophysics for 14 participants with ASD and 25 NTs. We found no evidence of reliable differences in eye tracking metrics during psychophysics between groups (Mann-Whitney tests, Z-values < 0.63, uncorrected p-values > 0.5). Eye tracking metrics were also not correlated with psychophysical suppression measures (SI at 98% or 3% contrast) across participants ($|r_{37} < 0.28|$, uncorrected p-values > 0.091).

Eye tracking data were obtained during fMRI for 14 participants with ASD and 24 NTs. The drift corrected mean and *SD* of distance from fixation were numerically higher for ASD vs. NT participants, but these differences were not statistically significant (Mann-Whitney tests, Z-values < 1.59, uncorrected p-values > 0.11). There was also no reliable difference in fixation time between groups (Z = 0.5, p = 0.6). Of the participants for whom eye tracking data were collected during fMRI, foveal MT ROIs were identified for 12 individuals with ASD and 19 NTs. Eye tracking metrics were not significantly correlated with fMRI suppression within hMT+ (at 98% or 3% contrast) across participants ($|r_{29} < 0.33|$, uncorrected p-values > 0.071). Although the conclusions that may be drawn from our eye tracking data are limited by the small sample size, we do not find evidence to suggest that weaker spatial suppression in ASD may be explained by

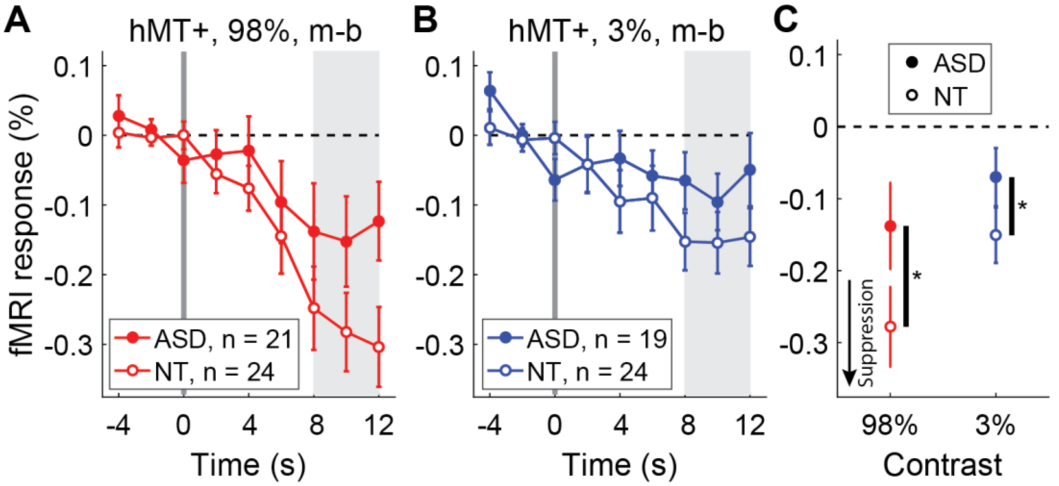

Supplementary Figure 6. FMRI results with blocks excluded for excess head motion and poor task performance. **A)** As in Figure 2, fMRI responses in foveal hMT+ to an increase in stimulus size; at time = 0 s, high contrast drifting gratings increased in size from medium (m) to big (b). **B)** The same, but for low contrast gratings. **C)** Average fMRI responses for each group (from shaded regions in **A** & **B**). Dots show group means; error bars are *S.E.M.* Asterisks indicate a significant main effect of group (ANOVA) at p = 0.049.

gross systematic differences in fixation behavior during psychophysical or fMRI task performance.

Finally, we compared MRS data quality metrics between ASD and NT participants, to determine whether differences in data quality may have contributed to the observed pattern of MRS results. We found that data quality was comparable overall between the two groups; we did not see significant differences between ASD and NT participants in frequency variability of water throughout the scan, number of TRs rejected for artifacts during frequency correction, or Glx fit residuals in either hMT+ or EVC (Mann-Whitney tests, Z-values < 1.53, uncorrected p-values > 0.126). The residual signal for GABA in hMT+ after fitting was higher for ASD (median = 5.2) versus NT participants (median = 4.7, Mann-Whitney test, $Z = 2.21$, uncorrected $p = 0.027$), and the spectral width of the creatine signal (FWHM) in EVC was broader for ASD participants (median = 8.7 Hz) versus NTs (8.3 Hz; Mann-Whitney test, $Z = 2.48$, uncorrected $p = 0.013$), suggesting somewhat lower data quality in participants with ASD on these metrics. However, neither of these were significant following correction for multiple comparisons (FDR corrected $p = 0.24$ and $p = 0.13$, respectively). There were no significant group differences for GABA residuals in EVC or creatine FWHM in hMT+ (Mann-Whitney tests, Z-values < 1.59, uncorrected p-values > 0.112). Thus, we do not find strong evidence to suggest that systematic differences in MRS data quality between groups may have greatly impacted the observed pattern of results.

Supplementary Methods

Supplementary Table 1 summarizes the missing or excluded data points from both participant groups across all experiments. Details for the exclusion procedures are provided in the relevant sections of the Methods in the main text.

Supplementary Table 1. Summary of missing or excluded data. The number of individuals with missing data is reported for each group.

Data type	ASD (total n = 28)	NT (total n = 35)
Psychophysics:		
Missing: thresholds for the smallest stimulus size	5	5
Excluded: catch trial accuracy < 80%†	1	2
Functional MRI:		
Missing: hMT+ data‡	3	10
Excluded: all fMRI data	1	0
Excluded: EVC data	4	1
MR Spectroscopy:		
Excluded: all MRS data	1	0

† Individuals excluded for poor catch trial performance were excluded from all analyses (psychophysics, fMRI, and MRS). These individuals are not included in the total sample sizes reported in the first row.

‡ We were unable to identify voxels in hMT+ with significant foveal selectivity (center > surround) in these participants.

Our computational approach is a direct application of the normalization model published by Reynolds and Heeger[20]. We have previously used a similar modeling technique to describe motion duration threshold data across a variety of experimental conditions in NT participants[5]. A summary of the current model approach is provided in the Methods; a thorough description of the method is provided below.

The core of the model, summarized in Equation 1, is reprinted here as:

$$R(x,\Theta,c) = \frac{E(x,\Theta,c) \times M(x,\Theta,M_g)}{S(x,\Theta,c) \times S_g + \sigma} \tag{1}$$

where R is the predicted model response as a function of stimulus space (x), orientation (Θ), and contrast (c). The suppressive gain parameter S_g is a scalar that controls the strength of divisive normalization[11], and σ is the semi-saturation constant, which controls the non-linearity of the predicted response as a function of stimulus contrast, as well as preventing the value of R from being undefined when S equals zero.

The parameters E, S, and M are each 2-dimensional representations of a population of computational processes, with selectivity across a spatial dimension (x) and an orientation dimension (Θ). The magnitudes of E and S are a function of stimulus contrast (c). Thus:

$$E(x,\Theta,c) = e(x_{w\_e},\Theta_{w\_e}) * N(x,\Theta,c) \tag{2}$$

where N is the stimulus image, which is a population-level representation of the stimulus input for the model, and $*$ denotes convolution. Specifically, N is a 2-dimensional Gaussian function whose amplitude is set by c. The widths of N in the x and Θ dimensions are determined by the shape of the stimulus being modeled. Likewise, e is a 2-dimensional Gaussian function, where the spatial and orientation selectivity for the excitatory drive (E) are determined by the width of spatial (x_{w_e}) and orientation (Θ_{w_e}) Gaussian parameters, referred to as excitatory spatial filters and orientation filters, respectively. These filter parameters can be thought of as the model equivalent of the spatial and orientation selectivity of neural receptive fields in visual cortex. The amplitude of e is set to 1, such that c determines the amplitude of E.

As with the stimulus parameter N, the top-down gain modulation field parameter M is a 2-dimensional Gaussian function: $M(x, \Theta, M_g)$. The center and width of top-down modulation in the spatial (x) and orientation (Θ) dimensions are determined by the mean and tuning width of $M(x)$ and $M(\Theta)$, while the amplitude is set by the top-down modulation gain factor M_g. The minimum value of M is set to 1, such that there is no effect of multiplying E x M (in the numerator of Supplementary Equation 1) outside of the region where top-down processing is focused (see Figure 4, cyan box).

The suppressive drive S is a function of both E and A, such that:

$$S(x,\Theta,c) = s(x_{w\_s},\Theta_{w\_s}) * \left(E(x,\Theta,c) \times M(x,\Theta,M_g)\right) \tag{3}$$

where s is another 2-dimensional Gaussian, like e in Supplementary Equation 2. Importantly, normalization models[20, 21] generally assume that the selectivity of s (in space; x_{w_s}) is broader than e. This produces the characteristic size tuning in the predicted model response, such that increasing stimulus size leads to a peak and then a decrease in the predicted response (see Figure 2F from our recent paper[5] for a graphical depiction of size tuning from this model).

The predicted motion duration threshold is given by Equation 2, reprinted here:

$$T = \frac{C}{R_{\text{peak}}} \tag{4}$$

where T is the predicted threshold in arbitrary units, and C is the criterion response level for the model. The value of R_{peak} is an average of the region surrounding the peak of the predicted model response R, such that:

$$R_{\text{peak}} = mean\big(R(x_{\max} - r_w : x_{\max} + r_w \,, \Theta_{\max} - r_w : \Theta_{\max} + r_w \,, c)\big) \tag{5}$$

where $R(x_{\max}, \Theta_{\max})$ is the maximum value of R, which happens to correspond to the center of the modeled stimulus. The parameter r_w determines the width of the region surrounding the peak in which the average is taken. In essence, using Supplementary Equation 5, we find the mean response in the region of the peak predicted response. While there is empirical support from electrophysiology in non-human primates for the notion that motion duration thresholds depend on the response of neurons whose spatial receptive field is centered on the stimulus[22], we note that this method differs slightly from our previous work[5], in which the threshold T depended strictly on the maximum of the predicted response R. Values for each model parameter across all three model variants (from Figure 5) are provided in Supplementary Table 2, and are adapted from our previous work[5].

By reducing the amplitude of the suppressive gain term S_g by 25% (i.e., from 1 to 0.75; Supplementary Table 2, Figure 4, Figure 5A-C), we sought to model weaker divisive normalization using the method described by Rosenberg and colleagues[11]. This 25% reduction in normalization strength is directly comparable to their published work (see their SI Appendix, pg. 2). Our two divisive normalization models are quite similar overall. One difference worth noting is that their model includes a first level consisting of model simple cells that are sensitive to spatial phase, and a second level of model complex cells for which divisive normalization is implemented, whereas our model does not include a phase-sensitive layer.

By adjusting the size of x_{w_e} from 4 to 5 (Supplementary Table 2, Figure 4, Figure 5D-E), we sought to model the effect of larger excitatory spatial filters on motion discrimination using an

Supplementary Table 2. Model parameters. Parameters marked with an asterisk (*) varied across the three model versions in Figure 5. Top-down spatial width values in parentheses are from the model variants shown in Supplementary Figure 4. Inf. = infinite.

**Parameter name**	**Weak norm. model**	**Large excitatory SF model**	**Narrow top-down model**
Stimulus contrast	0.03 or 0.98	0.03 or 0.98	0.03 or 0.98
Stimulus spatial center (x, a.u.)	0	0	0
Stimulus spatial width (a.u.)	1, 2, or 12	1, 2, or 12	1, 2, or 12
Stimulus orientation (Θ, °)	-90	-90	-90
Stimulus orientation width (°)	3	3	3
*Excitatory spatial pooling width (x_{w_e}, a.u.)	4	5 vs. 4	4
Excitatory orientation pooling width (Θ_{w_e}, °)	25	25	25
Top-down spatial center (x, a.u.)	0	0	0
*Top-down spatial width (a.u.)	14	14	6 vs. 14 (1 or 2 vs. 6)
Top-down orientation (Θ, °)	-90	-90	-90
Top-down orientation width (°)	Inf.	Inf.	Inf.
Top-down gain (M_g)	4	4	4
Suppressive spatial pooling width (x_{w_s}, a.u.)	40	40	40
Suppressive orientation pooling width (Θ_{w_s}, °)	25	25	25
*Suppressive gain (S_g)	0.75 vs. 1	1	1
Semi-saturation constant (σ, a.u.)	0.0002	0.0002	0.0002
Criterion (C, a.u.)	600	600	600
Response region width (r_w, a.u.)	1	1	1

approach comparable to that of Schauder and colleagues[12]. This difference in excitatory spatial filter size (25% larger for ASD; see their Figure 2) is in line with the increased model RF size reported in their study. While generally comparable, a key difference between our normalization model and their divisive center-surround model is that their model includes separate parameters for the contrast sensitivity of the excitatory and suppressive drive (see their Equations 3 & 4). In our model, the contrast sensitivity of the excitatory and suppressive drive is the same, but the latter is simply more broadly tuned in space.

We used linear mixed effects modeling in our post-hoc analyses that controlled for age, sex, and non-verbal IQ. With the addition of these 3 factors, we chose to use linear mixed effects models rather than ANOVAs, in order to make our analyses robust against missing factor combinations (i.e., rank deficiency). Linear mixed effects models were fit using a maximum likelihood procedure via the *fitlme.m* function in MATLAB. For our control analyses, we used Mann-Whitney in cases where data were not normally distributed.

To account for possible residual effects of head motion in our fMRI results, we performed control analyses in which we excluded individual fMRI blocks (10 s) in individual participants in which excessive head motion was detected. Although we performed motion correction in BrainVoyager (Brain Innovation, Maastricht, Netherlands) as part of our fMRI preprocessing, such corrections are incomplete at best, and large head movements may still negatively affect data quality even after correction. Our motion correction procedure yielded estimates of translation in x, y, and z dimensions, as well as roll, pitch, and yaw rotations between each pair of subsequent TRs. Framewise displacement[23] was calculated by taking the sum of the absolute value of the displacement in each of these six dimensions, with rotation converted from degrees to millimeters on the surface of a sphere with a radius of 50 mm. The threshold for excessive head motion was defined as a framewise displacement > 0.9 mm using a pre-defined criterion[24]. We excluded all fMRI blocks that contained TRs with any framewise displacement values larger than this threshold. In order to account for the slow time course of the hemodynamic response, framewise displacement data were scrutinized within a time window spanning from 16 s before to 4 s after each 10 s fMRI block.

We also sought to account for possible effects of task disengagement during fMRI by excluding individual fMRI scans in which the participant performed poorly on the fixation task. The task consisted of a color-shape conjunction search, in which the participant responded with a button press to the appearance of a green circle in a series of small, briefly presented colored shapes. Poor task performance was defined based on a post-hoc threshold of 60% hit rate; we excluded all fMRI scans (4 min) in which performance was below this threshold.

Eye tracking data were acquired during both psychophysics and fMRI using an SR Research (Ottawa, Canada) EyeLink 1000 infrared eye tracking camera system. Data were acquired at a sampling rate of 1000 Hz.

We identified fixation periods using a sliding window analysis along with post-hoc drift correction. Specifically, we used a dispersion-based fixation detection algorithm[25, 26]. Fixations were defined within set periods of at least 100 ms for which the maximum distance of any gaze position measurement within the set compared to the set's centroid does not exceed a threshold radius of 1°. In this algorithm, subsequent time points were added to a set until this distance threshold was exceeded, at which point a new set was defined. The parameters that defined fixation periods were taken from previous work using this method[26]. Next, post-hoc drift correction was performed by calculating the average gaze position across all fixations within a 10 s period, taking the difference between this average fixation position and the intended fixation position (i.e., the fixation mark at the center of the screen), and subtracting this value from all gaze position measures within the 10 s period. This analysis assumes that participants understood the instructions to maintain fixation at the center of the screen and were attempting to do so throughout the task. We further assumed that systematic differences between measured and instructed gaze position may be accounted for by drift in the eye tracking camera calibration over time (e.g., due to slight postural changes). We believe these assumptions are reasonable given that fixation performance was monitored by study staff, who verified comprehension of the fixation protocol with the participants prior to task initiation. This drift correction method is a subtractive version of an algorithm developed by Vadillo and colleagues[27]. We chose a subtractive method, rather than a multiplicative one (as used in their original paper), as we found the former was better suited to drift correction during a central fixation task, and did not

dramatically alter the magnitude of gaze position variability metrics.

We calculated three different eye tracking metrics: mean distance from center (a measure of gaze accuracy in space), the standard deviation of fixation distance from center (a spatial measure of gaze variability), and mean fixation duration (a measure of fixation stability across time). Because we found that these data were not normally distributed, we used Mann-Whitney tests to assess group differences, and Spearman’s Rho to test for correlations.

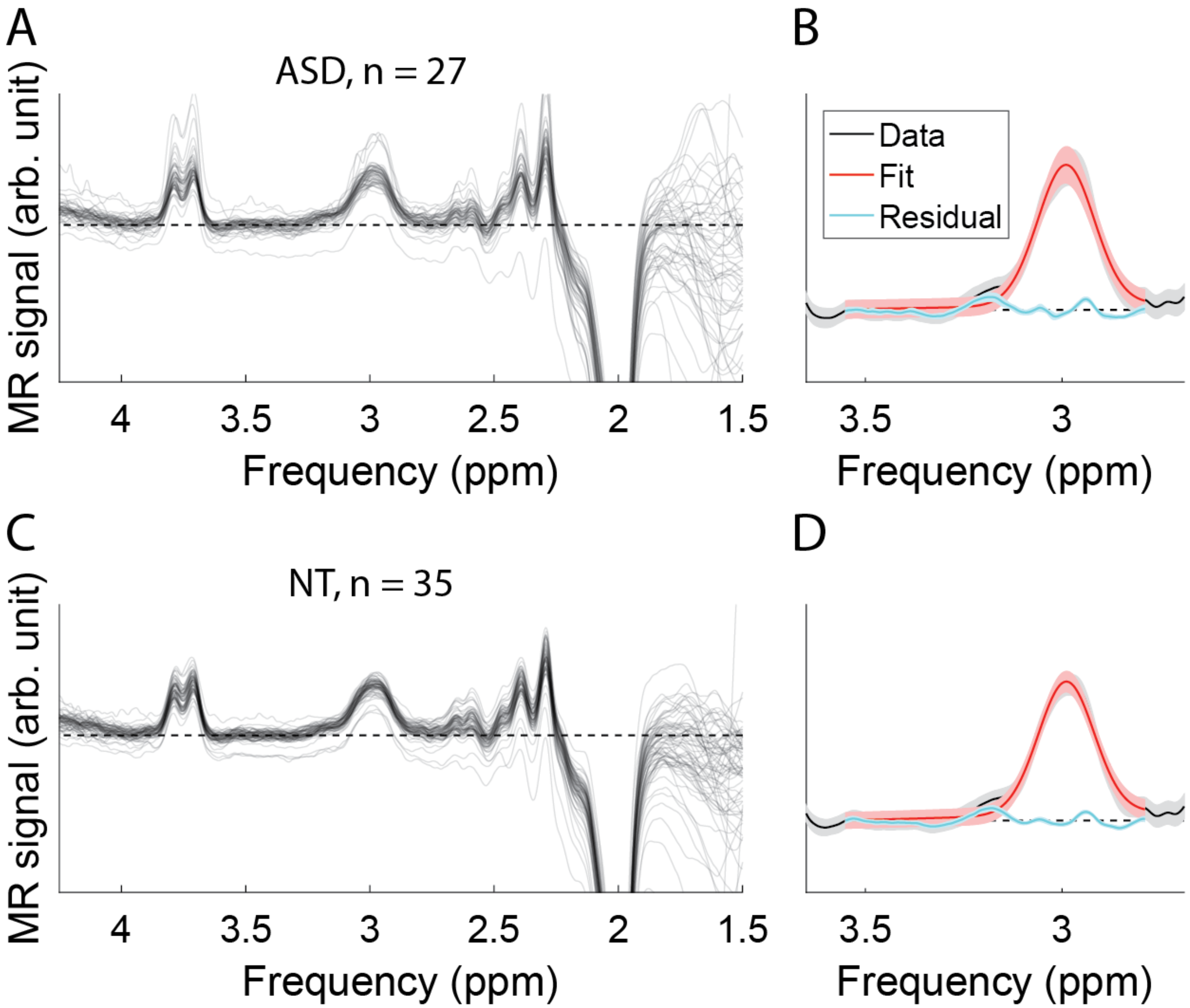

Supplementary Figure 7. MR spectra from hMT+. **A)** MR spectroscopy data from n = 27 participants with ASD. Signal intensity is shown in arbitrary units. **B)** GABA+ peak in ASD. Data are shown in black, Gaussian fits are shown in red, residuals are shown in blue. Panels **C** & **D** show the same, but for n = 35 NT participants. Lines show means; shaded areas show 1 *SD*. Axes are scaled equally in panels **A** & **C**, and in panels **B** & **D**.

Supplementary References

1. Rubenstein JLR, Merzenich MM. Model of autism: Increased ratio of excitation/inhibition in key neural systems. *Genes, Brain and Behavior* **2**, 255-267 (2003).

2. Yizhar O*, et al.* Neocortical excitation/inhibition balance in information processing and social dysfunction. *Nature* **477**, 171-178 (2011).

3. Foss-Feig JH*, et al.* Searching for cross-diagnostic convergence: Neural mechanisms governing excitation and inhibition balance in schizophrenia and autism spectrum disorders. *Biological Psychiatry* **81**, 848-861 (2017).

4. Foss-Feig JH, Tadin D, Schauder KB, Cascio CJ. A substantial and unexpected enhancement of motion perception in autism. *The Journal of Neuroscience* **33**, 8243-8249 (2013).

5. Schallmo M-P*, et al.* Suppression and facilitation of human neural responses. *eLife* **7**, e30334 (2018).

6. Mullins PG*, et al.* Current practice in the use of MEGA-PRESS spectroscopy for the detection of GABA. *NeuroImage* **86**, 43-52 (2014).

7. Shushruth S, Mangapathy P, Ichida JM, Bressloff PC, Schwabe L, Angelucci A. Strong recrrent networks compute the orientation tuning of surround modulation in the primate primary visual cortex. *The Journal of Neuroscience* **32**, 308-321 (2012).

8. Sato TK, Haider B, Haüsser M, Carandini M. An excitatory basis for divisive normalization in visual cortex. *Nature Neuroscience*, 1-3 (2016).

9. Fatemi SH. The hyperglutamatergic hypothesis of autism. *Progress in Neuro-Psychopharmacology and Biological Psychiatry* **32**, 911 (2008).

10. Brown MS, Singel D, Hepburn S, Rojas DC. Increased glutamate concentration in the auditory cortex of persons with autism and first-degree relatives: A 1H-MRS study. *Autism Research* **6**, 1-10 (2013).

11. Rosenberg A, Patterson JS, Angelaki DE. A computational perspective on autism. *Proceedings of the National Academy of Sciences* **112**, 9158-9165 (2015).

12. Schauder KB, Park WJ, Tadin D, Bennetto L. Larger receptive field size as a mechanism underlying atypical motion perception in autism spectrum disorder. *Clinical Psychological Science* **5**, 827-842 (2017).

13. Sysoeva OV, Galuta IA, Davletshina MS, Orekhova EV, Stroganova TA. Abnormal size-dependent modulation of motion perception in children with autism spectrum disorder (ASD). *Frontiers in Neuroscience* **11**, (2017).

14. Betts LR, Taylor CP, Sekuler AB, Bennett P. Aging reduces center-surround antagoinism in visual motion processing. *Neuron* **45**, 361-366 (2005).

15. Betts LR, Sekuler AB, Bennett PJ. Spatial characteristics of center-surround antagonism in younger and older adults. *Journal of Vision* **9**, 25-25 (2009).

16. Betts LR, Sekuler AB, Bennett PJ. Spatial characteristics of motion-sensitive mechanisms change with age and stimulus spatial frequency. *Vision Research* **53**, 1-14 (2012).

17. Murray SO*, et al.* Sex differences in visual motion processing. *Current Biology* **28**, 2794-2799 (2018).

18. Melnick MD, Harrison BR, Park S, Bennetto L, Tadin D. A strong interactive link between sensory discriminations and intelligence. *Current Biology* **23**, 1013-1017 (2013).

19. Arranz-Paraiso S, Serrano-Pedraza I. Testing the link between visual suppression and intelligence. *PLOS ONE* **13**, e0200151 (2018).

20. Reynolds JH, Heeger DJ. The normalization model of attention. *Neuron* **61**, 168-185 (2009).

21. Heeger DJ. Normalization of cell responses in cat striate cortex. *Visual Neuroscience* **9**, 181-197 (1992).

22. Liu LD, Haefner RM, Pack CC. A neural basis for the spatial suppression of visual motion perception. *eLife* **5**, e16167 (2016).

23. Power JD, Barnes KA, Snyder AZ, Schlaggar BL, Petersen SE. Spurious but systematic correlations in functional connectivity MRI networks arise from subject motion. *NeuroImage* **59**, 2142-2154 (2012).

24. Siegel JS*, et al.* Statistical improvements in functional magnetic resonance imaging analyses produced by censoring high-motion data points. *Human Brain Mapping* **35**, 1981-1996 (2014).

25. Anliker L. Eye movements: On-line measurement, analysis, and control. In: *Eye movements and psychological processes* (eds: Monty RA, Senders JW). Erlbaum (1976).

26. Blignaut P. Fixation identification: The optimum threshold for a dispersion algorithm. *Attention, Perception, & Psychophysics* **71**, 881-895 (2009).

27. Vadillo MA, Street CNH, Beesley T, Shanks DR. A simple algorithm for the offline recalibration of eye-tracking data through best-fitting linear transformation. *Behavior Research Methods* **47**, 1365–1376 (2015).
