# Supplementary material for: Weaker neural suppression in autism
Source: Nat Commun. 2020 May 29;11:2675. doi: 10.1038/s41467-020-16495-z (PMC7260360; doi:10.1038/s41467-020-16495-z)
Supplement: Supplementary file 3 — Reporting Summary [file 41467_2020_16495_MOESM3_ESM.pdf]

nature research

Corresponding author(s): Michael-Paul Schallmo

Last updated by author(s): Apr 28, 2020

Reporting Summary

Nature Research wishes to improve the reproducibility of the work that we publish. This form provides structure for consistency and transparency in reporting. For further information on Nature Research policies, see Authors & Referees and the Editorial Policy Checklist .

Statistics

For all statistical analyses, confirm that the following items are present in the figure legend, table legend, main text, or Methods section.

n/a	Confirmed	
☐	☒	The exact sample size (n) for each experimental group/condition, given as a discrete number and unit of measurement
☐	☒	A statement on whether measurements were taken from distinct samples or whether the same sample was measured repeatedly
☐	☒	The statistical test(s) used AND whether they are one- or two-sided<br>*Only common tests should be described solely by name; describe more complex techniques in the Methods section.*
☐	☒	A description of all covariates tested
☐	☒	A description of any assumptions or corrections, such as tests of normality and adjustment for multiple comparisons
☐	☒	A full description of the statistical parameters including central tendency (e.g. means) or other basic estimates (e.g. regression coefficient) AND variation (e.g. standard deviation) or associated estimates of uncertainty (e.g. confidence intervals)
☐	☒	For null hypothesis testing, the test statistic (e.g. F, t, r) with confidence intervals, effect sizes, degrees of freedom and P value noted<br>*Give P values as exact values whenever suitable.*
☒	☐	For Bayesian analysis, information on the choice of priors and Markov chain Monte Carlo settings
☒	☐	For hierarchical and complex designs, identification of the appropriate level for tests and full reporting of outcomes
☐	☒	Estimates of effect sizes (e.g. Cohen's d, Pearson's r), indicating how they were calculated

Our web collection on statistics for biologists contains articles on many of the points above.

Software and code

Policy information about availability of computer code

Data collection	Presentation (version 14.9), MATLAB (version R2015a), PsychToolbox (version 3.0.11), Palamedes toolbox (version 1.7.0), MoTrak (version 1.0.3.4), Philips iViewBOLD (Achieva)
Data analysis	MATLAB (version R2016a), Palamedes toolbox (version 1.7.0), BrainVoyager QX (version 2.8), MATLAB code for our implementation of the normalization model is available at: github.com/mpschallmo/WeakerNeuralSuppressionAutism

For manuscripts utilizing custom algorithms or software that are central to the research but not yet described in published literature, software must be made available to editors/reviewers. We strongly encourage code deposition in a community repository (e.g. GitHub). See the Nature Research guidelines for submitting code & software for further information.

Data

Policy information about availability of data

All manuscripts must include a data availability statement. This statement should provide the following information, where applicable:

- Accession codes, unique identifiers, or web links for publicly available datasets
- A list of figures that have associated raw data
- A description of any restrictions on data availability

Data from this study are available from: nda.nih.gov/edit_collection.html?id=2266
The following figures have associated raw data: Figures 2, & 3, Supplementary Figures 2, 3, 6, & 7.

Field-specific reporting

Please select the one below that is the best fit for your research. If you are not sure, read the appropriate sections before making your selection.

Life sciences study design

All studies must disclose on these points even when the disclosure is negative.

Sample size	Sample sizes for each subject group (ASD & NT) were chosen based on current standards from recently published studies in this field (e.g., Manning et al., 2015, J. Neurosci.; Robertson et al., 2016, Curr. Bio.; Schauder et al., 2017, Clin. Psy. Sci.)
Data exclusions	Supplementary Table 1 summarizes the missing or excluded data points from both participant groups across all experiments. Details for the exclusion procedures are provided in the relevant sections of the Methods in the main text, including whether exclusion criteria were pre-established or post-hoc.
Replication	Successful repeated measurements were taken within and across subjects (n = 28 with ASD, n = 35 NTs) for each experiment, as described in the methods.
Randomization	Subjects were divided into experimental groups based on ASD status, as described in the methods. We conducted post-hoc analyses to examine group differences in duration thresholds, size indices, and fMRI suppression, with age, sex, and non-verbal IQ included as covariates, as described in the Supplemental Information.
Blinding	Experimenters were not blinded to subject group status (i.e., ASD vs. NT). Blinding is not standard procedure in observational brain imaging experiments, and is not expected to have a significant impact on data collection.

Reporting for specific materials, systems and methods

We require information from authors about some types of materials, experimental systems and methods used in many studies. Here, indicate whether each material, system or method listed is relevant to your study. If you are not sure if a list item applies to your research, read the appropriate section before selecting a response.

Materials & experimental systems

n/a	Involved in the study	
☒	☐	Antibodies
☒	☐	Eukaryotic cell lines
☒	☐	Palaeontology
☒	☐	Animals and other organisms
☐	☒	Human research participants
☒	☐	Clinical data

Methods

n/a	Involved in the study	
☒	☐	ChIP-seq
☒	☐	Flow cytometry
☐	☒	MRI-based neuroimaging

Human research participants

Policy information about studies involving human research participants

Population characteristics	Population characteristics are described in Table 1 of the manuscript. The following demographic factors did not differ significantly between the two participant groups: age, biological sex, non-verbal IQ (from the Wechsler Abbreviated Scale of Intelligence; WASI), and handedness.
Recruitment	Subjects were recruited through advertisements in the community (e.g., message boards, email lists), and by re-contacting previous research participants. As noted in the Discussion, the individuals with ASD who participated in our study were generally high-functioning, as reflected in their relatively high non-verbal IQ scores (Table 1). This may reflect self-selection bias, and may be a consequence of recruiting participants who are willing and able to take part in a demanding set of behavioral and neuroimaging experiments over the course of multiple days.
Ethics oversight	All procedures were approved by the Institutional Review Board at the University of Washington and conformed to the guidelines for research on human subjects from the Declaration of Helsinki.

Note that full information on the approval of the study protocol must also be provided in the manuscript.

Magnetic resonance imaging

Experimental design

Design type	Block design task
Design specifications	Our fMRI paradigm was designed to measure spatial suppression and has been described previously (Schallmo et al., 2018). In this task, smaller (2° diameter) and larger (12°) drifting gratings were presented at the center of the screen in alternating 10 s blocks. Grating duration was 400 ms; inter-stimulus interval (ISI) was 225 ms. There were 16 gratings in each block, which drifted in 1 of 8 possible directions (order randomized and counterbalanced). A single fMRI scanning run (4.2 min long) included a total of 25 blocks (13 smaller, 12 larger). Stimulus contrast was either 3% or 98% in

separate runs. No baseline or rest blocks were included; stimuli appeared within the central 2° in all blocks. Previous studies have used this type of alternating block design to measure surround suppression in early visual cortex using fMRI. We chose this paradigm to measure fMRI suppression in ASD for its simplicity and because it allowed us to easily exclude particular blocks from analysis. Each participant completed 2-4 runs at each contrast level across 1 or 2 scanning sessions (some participants chose to end the experiment early, e.g., due to fatigue).

Our fMRI experiment also included two functional localizer scans, which were used to identify regions of interest (ROIs). The first localizer was designed to identify the motion-selective brain area known as human MT complex (hMT+; Supplemental Figure 1A). We refer to this area as hMT+ to indicate that we did not attempt to differentiate regions MT and MST. This localizer consisted of alternating 10 s blocks of drifting and static gratings (2° diameter, 15% contrast; Supplemental Figure 1B). There were 25 blocks total (13 static, 12 drifting). Grating duration was 400 ms with a 225 ms ISI. The second localizer scan was used to identify voxels with retinotopic selectivity for the central 2°. Using a differential localizer approach allowed us to identify voxels that responded more strongly to stimuli in the center vs. surrounding portion of the screen. This scan consisted of alternating 10 s blocks of phase-reversing checkerboards (8 Hz; 100% contrast) within the central 2°, or within an annular region from 2-12° eccentricity (Supplemental Figure 1C & D). There were 16 blocks in the second localizer scan (8 center, 8 annulus). Rest blocks were not included during either localizer. Participants performed the same fixation task as in the main fMRI experiment during both localizers. One run of each localizer type was included in each scanning session.

Behavioral performance measures

Correct button presses and response time were recorded. The task consisted of a color-shape conjunction search, in which the participant responded with a button press to the appearance of a green circle in a series of small, briefly presented colored shapes. Poor task performance was defined based on a threshold of 60% hit rate; for our control analysis, we excluded all fMRI scans (4 min) in which performance was below this threshold, as described in the Supplementary Information.

Acquisition

Imaging type(s)

Functional, structural

Field strength

3 tesla

Sequence & imaging parameters

Each scanning session began with a T1-weighted anatomical scan (1 mm isotropic resolution), followed by whole-brain gradient echo EPI (3 mm isotropic resolution, 30 oblique-axial slices with a 0.5 mm gap, 2 s repetition time [TR], 25 ms echo time [TE], 79° flip angle, anterior-posterior phase encoding direction). A single run with the opposite phase encoding direction (posterior-anterior; 3 TRs) was also acquired during each scanning session, to facilitate geometric distortion compensation.

Area of acquisition

whole brain

Diffusion MRI ☐ Used ☒ Not used

Preprocessing

Preprocessing software

BrainVoyager QX (version 2.8)

Normalization

None; all analyses were within-subject ROI-based

Normalization template

Not normalized

Noise and artifact removal

We used BrainVoyager for motion correction (trilinear detection and sinc interpolation), geometric distortion compensation (COPE plugin, v0.5), and high-pass filtering (> 2 cycles / scan).

Volume censoring

Our motion correction procedure yielded estimates of translation in x, y, and z dimensions, as well as roll, pitch, and yaw rotations between each pair of subsequent TRs. Framewise displacement was calculated by taking the sum of the absolute value of the displacement in each of these six dimensions, with rotation converted from degrees to millimeters on the surface of a sphere with a radius of 50 mm. The threshold for excessive head motion was defined as a framewise displacement > 0.9 mm. For our control analyses, we excluded all fMRI blocks that contained TRs with any framewise displacement values larger than this threshold. In order to account for the slow time course of the hemodynamic response, framewise displacement data were scrutinized within a time window spanning from 16 s before to 4 s after each 10 s fMRI block, as described in the Supplementary Information.

Statistical modeling & inference

Model type and settings

ANOVAs with subjects treated as a random variable and nested within groups

Effect(s) tested

The following effects were tested: group (ASD vs. NT), stimulus size (small, medium, big -- motion duration thresholds only), stimulus contrast (3 vs. 98%)

Specify type of analysis: ☐ Whole brain ☒ ROI-based ☐ Both

Anatomical location(s)

ROIs were defined based on fuctional localizer activation maps at the individual subject level. ROIs were defined in each hemisphere in the space of the functional data using a standard correlational analysis. Bilateral ROIs were defined for area hMT+ in the lateral occipital lobe (Supplementary Figure 1A &B, Supplementary Figure 2A & B) from the motion vs. static functional localizer data, and for EVC near the occipital pole from the center vs. surround functional localizer data. ROIs in hMT+ were further refined by

finding voxels within hMT+ that additionally showed significant retinotopic selectivity for the central 2° in the center vs. surround functional localizer (1-tailed $p < 0.05$; Supplementary Figure 1C & D). Thus, the hMT+ ROIs were identified from the intersection of voxels in the lateral occipital lobe showing selectivity for motion > static, and center > surround.

Statistic type for inference
(See Eklund et al. 2016)

Voxel-wise

Correction

Bonferroni

Models & analysis

n/a	Involved in the study
☒	☐ Functional and/or effective connectivity
☒	☐ Graph analysis
☒	☐ Multivariate modeling or predictive analysis
